# Isolation of *Escherichia coli* 0157:H7 Strain from Fecal Samples of Zoo Animal

**DOI:** 10.1155/2013/843968

**Published:** 2013-12-30

**Authors:** Aseel Mohammed Hamzah, Aseel Mohammed Hussein, Jenan Mahmoud Khalef

**Affiliations:** Zoonotic Diseases Unit, Veterinary Medicine College, Baghdad University, Iraq

## Abstract

The isolation and characterization of *Escherichia coli* O157:H7 strains from 22 out of 174 fecal samples from petting zoo animals representing twenty-two different species (camel, lion, goats, zebra, bear, baboon monkey, Siberian monkey, deer, elk, llama, pony, horses, fox, kangaroo, wolf, porcupine, chickens, tiger, ostrich, hyena, dogs, and wildcats) were investigated. One petting Al-Zawraa zoological society of Baghdad was investigated for *E. coli* O157:H7 over a 16-month period that spanned two summer and two autumn seasons. Variation in the occurrence of *E. coli* O157:H7-positive petting zoo animals was observed, with animals being culture positive only in the summer months but not in the spring, autumn, or winter. *E. coli* O157:H7 isolates were distinguished by agglutination with *E. coli* O157:H7 latex reagent (Oxoid), identified among the isolates, which showed that multiple *E. coli* strains were isolated from one petting zoo animal, in which a single animal simultaneously shed multiple *E. coli* strains; *E. coli* O157:H7 was isolated only by selective enrichment culture of 2 g of petting zoo animal feces. In contrast, strains other than O157:H7 were cultured from feces of petting zoo animals without enrichment.

## 1. Introduction

Since it was first identified in the early 1980s [1], the enterohemorrhagic *Escherichia coli* (EHEC) strains are a subset of Shiga toxin-producing *E. coli *strains that have been associated with animals and human diseases. In humans including self-limited watery diarrhea, hemorrhagic colitis, and the hemolytic-uremic syndrome (HUS) [2], this syndrome happened in 2–7% of people with *E. coli* 0157:H7 infection causing bloody diarrhea [3], in many areas of the world [4–8]. Among the EHEC serotypes, O157:H7, which expresses somatic (O) antigen 157 and flagellar (H) antigen 7, causes serious morbidity and large disease outbreaks, making this bacterium one of the most important food-borne and waterborne pathogens worldwide [9–11]. In 1995, an outbreak of *E. coli *O157:H7 infections in people was traced to jerky made from deer meat [12].

The vulnerable sectors of the community (children and the elderly) are at the most risk of developing severe infection, making it a very emotive issue in public health and across the food and agricultural industries [13–17].

Cattle appear to be major reservoir for verotoxin-producing *E. coli* O157 [18–22], although it has also been found in sheep, goats, heifer, birds, deer, geese, turkey, seabirds, dogs, cat, gull, chicken, pig, monkey, reptiles, llama, and horses, as well as on flies [23–30]. However, the extent to which these animal species play a role in the epidemiology of O157 infection remains to be established. Although most infections of O157 in humans have been linked to exposure to a food vehicle or water, person-to-person transmission of O157 and transmission by direct contact with animals or animal manure have also been reported [31].

Petting zoo visits are popular leisure activities and also have become an important feature of education for young children. Such visits are highly beneficial to children in helping them to learn about aspects of animal husbandry and farm produce. Close contact with the animals is often encouraged,such as petting and feeding animals, especially to the main group of visitors, young children, of acquiring severe zoonotic infections during visits to petting zoos. Several outbreak recorded of Escherichia coli O157 infections occurred among agricultural fair, festival, and petting zoo visitors in farm visits occurred in many country in Pennsylvania, Washington [32], Canada [33], and North Wales [34]. During 2003 to2004the capacity of STEC 0157 to persist and multiplicative in the farm environment (animal feces, straw, soil, water) [35] and their natural occurrence in several wild animal species from which interspecies transmission to domestic animals may occur [36], preventing the introduction of the infection, routine testing of brought-in replacement animals, culling infected animals, and closing infected petting zoos, all do not appear to be feasible or effective control measures.

Consistent with this, Pritchard et al. [37] found no obvious value in preentry of bacteriological testing of animals during a longitudinal study on a farm open to the public. *Escherichia coli* O157:H7 has been detected in the feces of white-tailed deer (*Odocoileus virginianus*), but the extent of direct or indirect zoonotic risk of this source of *E. coli* O157:H7 has yet to be determined [38].

The morbidity and the mortality associated with outbreaks of gastrointestinal illnesses caused by STEC have highlighted the threat they pose to public health. Therefore, monitoring the presence of *E. coli* in animals will assure prompt diagnosis and identify the source of infection that may assist in risk management.

Epidemiological investigation of O157 strain in animal populations has focused mainly on the bovine reservoir and recently in horses, so the prevalence in other animals is not well known in Iraq. The aim of this study was to determine the prevalence of *E. coli* O157 in fecal samples collected from zoo animals in the Al-Zawraa zoological society of Baghdad in Iraq.

## 2. Material and Methods

### 2.1. Animals

One hundred seventy-four fecal samples were collected from animals kept in Al_Zawraa zoological society of Baghdad of different breeds, ages, and sexes which were included in the study during the period from June 2010 to June 2013. Twenty-two animal species, representing, camel, lion, goats, zebra, bear, baboon monkey, Siberian monkey, deer, elk, llama, pony, horses, fox, kangaroo, wolf, porcupine, chickens, tiger, ostrich, hyena, dogs, and wildcats.

### 2.2. Sample Inoculation

Three g of each fecal sample was mixed with normal saline and centrifuged. The supernatant was discarded and the deposit was inoculated with 5 mL of buffered peptone water (Oxoid).

### 2.3. Selective Enrichment and Isolation of *E. coli* O157:H7

One loopful from cultured broth was placed in plate with Cefixime Tellurite-Sorbitol MacConkey (CT-SMAC) agar. The plate was streaked for isolation and incubated overnight. After incubation, all nonsorbitol-fermenting (grey/white) colonies were plated on both blood agar and Levine Eosin Methylene blue agar and incubated overnight at 37°C.

### 2.4. Agglutination Test

Individual isolates colonies of nonsorbitol-fermenting colonies on CT-SMAC medium were tested for the presence of the O157 and the H7 antigens by agglutination with *E. coli *O157 and H7 latex reagent (Oxoid).

## 3. Results

*E. coli *O157:H7 was isolated from twenty-two out of 174 fecal samples collected in Al_Zawraa zoological societies of Baghdad which are summarized in Table 1.* E. coli *O157:H7 was isolated from 30% bear, 22.2% deer, 28.6% pony, 16.7% horses, 20% zebra, 66.7% ostrich, 12.5% hyena, 16.7% llama, 25% goat, and 28.6% jaguar. The percentage of EHEC *E. coli* isolate and characteristic of *E. coli *O157:H7 are shown in Table 2.

EHEC strains were isolated from 50% bear, 33.3% deer, 14.3% pony, 4.55% lion, 8.3% horse, 100% ostrich, 11.1% camel, 16.7% llama, and 28.6% jaguar.

**Figure 1:**
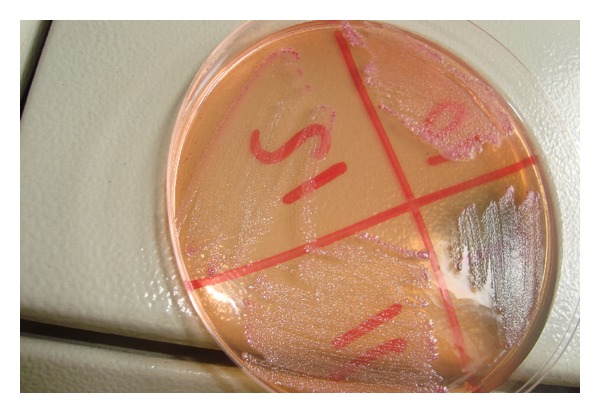
Sorbitol-nonfermented colonies of *E. coli *O157:H7 on CT-SMAC media.

The *E. coli* O157:H7 appears as sorbitol-nonfermented colonies on MacConkey agar (white-gray) as shown in Figure 1.

**Figure 2:**
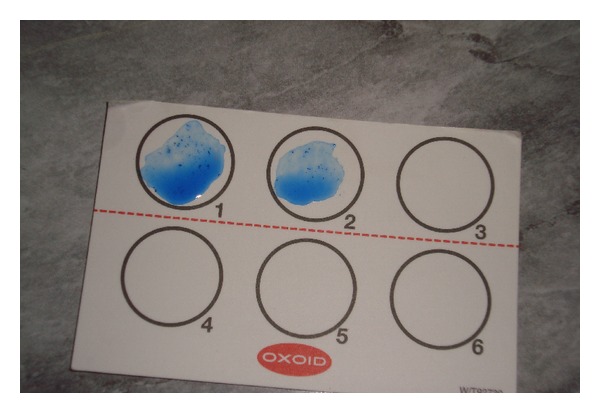
Agglutination of *E. coli *O157 and H7 latex reagent with isolates colonies of nonsorbitol-fermenting colonies on CT-SMAC medium.

**Figure 3:**
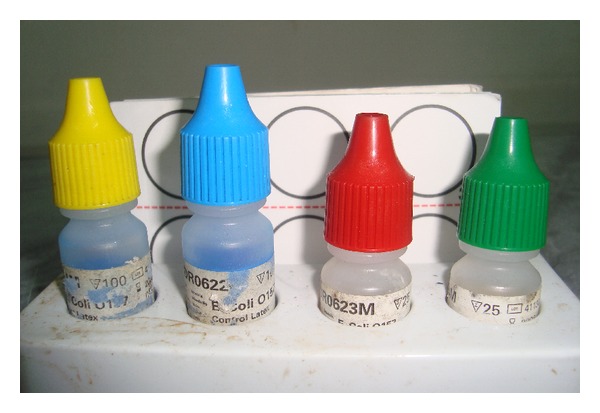
Agglutination kit (Oxoid) for diagnosis of *E. coli *O157:H7.

Individual isolates colonies of non-sorbitol-fermenting colonies on CT-SMAC medium were agglutinated with *E. coli* O157 and H7 latex reagent (Oxoid) as shown in Figures 2 and 3.

## 4. Discussion

This study includes the isolation of *E. coli* derived from twenty-two mammalian species from one zoo; this is the first report concerning the isolation of *E. coli* O157:H7 from petting zoo animals in Iraq.

Whereas ruminants are considered to be reservoir of *E. coli* O157:H7 infections, wild bird may play a key role in emergence by providing a “zoonotic pool” of the infectious agents mainly *E. coli* O157:H7; wild bird play an important role in dissemination of *E. coli* O157:H7; could be the main reservoir for *E. coli* O157:H7 especially gull that spreads *E. coli* O157:H7 to cattle and other animals [39, 40]. Other researcher [41] found that manure, rail and environmental of petting zoo animal that causes human infected cases with* E. coli* 0157 so that different isolated rate among animal species could be related with manure, animal food, water supply [11] contaminated with* E. coli* 0157 that may contaminated with feces of wild bird. The 100% carriage rate of *E. coli* O157:H7 in ostrich in this investigation is almost the same as that in cattle, which suggests that *E. coli* O157:H7 strains are probably widespread in ostrich populations. Because of the popularity of petting zoo, petting zoo animals with *E. coli* 0157:H7 have the potential to make large numbers of people ill.

Visitors had direct contact with animals that are potential sources of enteric pathogens. Visitors could eat and drink while interacting with animals, and, with the exception of strollers, there were no exclusions on items being brought into animal venues and visitors did not receive educational messages concerning disease risk and prevention measures.

There were insufficient hand washing signs and hand washing stations within the animal venues and midways.

Standards should outline the need for adequate hand washing facilities, appropriate disposal of manure, and proper cleaning of the environment, including rails and floors. Most of these animals did not carry *E. coli* 0157:H7 during the study period.

The seasonality of the incidence of *E. coli* O157:H7 in petting zoo animals, coupled with the increase in *E. coli* O157:H7 associated with food-borne illness during the summer months, suggests that environmental replication plays a key role in the epidemiology of infections [42, 43].

## Figures and Tables

**Table 1:** Number of positive *E. coli *O157:H7 animals from fecal samples collected and date of each collection for different animal species in Al-Zawraa zoological society of Baghdad.

Animal spp.	Number of samples	Month of sample	No. of positive animals	Percentage of positive animals for *E. coli *O157:H7
Bear	10	June	3	30%
Deer	9	June	2	22.2%
Pony	7	June	2	28.6%
Lion	22	April	3	13.6%
Elk	8	April	1	12.5%
Dog	8	September	Nil	0%
Horse	12	April	2	16.7%
Wildcat	5	September	Nil	0%
Zebra	5	September	1	20%
Siberia monkey	9	February	Nil	0%
Ostrich	3	June	2	66.7%
Baboon monkey	9	February	Nil	0%
Hyena	8	April	1	12.5%
Kangaroo	1	April	Nil	0%
Wolf	5	February	Nil	0%
Camel	9	June	Nil	0%
Fox	6	June	Nil	0%
Porcupine	5	February	Nil	0%
Llama	6	June	1	16.7%
Goat	8	June	2	25%
Jaguar	7	April	2	28.6%
Chicken	6	February	Nil	0%

**Table 2:** The percentage of EHEC *E. coli* isolate and *E. coli *O157:H7 with its characteristic features.

Animal spp.	Positive samples for EHEC	% of *E. coli* isolates	Positive sample for O157:H7	Sorbitol fermentation	Positive for O157 antigen	Positive for H7 antigen
Bear	5	50%	3	+	+	+
Deer	3	33.3%	2	+	+	+
Pony	1	14.3%	2	+	+	+
Lion	1	4.55%	3	+	+	+
Elk	Nil	—	1	+	+	+
Dog	Nil	—	Nil	−	−	−
Horse	1	8.3%	2	+	+	+
Wildcat	Nil	—	Nil	−	−	−
Zebra	Nil	—	1	+	+	+
Siberia monkey	Nil	—	Nil	−	−	−
Ostrich	3	100%	2	+	+	+
Baboon monkey	Nil	—	Nil	−	−	−
Hyena	Nil	—	1	+	+	+
Kangaroo	Nil	—	Nil	−	−	−
Wolf	Nil	—	Nil	−	−	−
Camel	1	11.1%	Nil	−	−	−
Fox	Nil	—	Nil	−	−	−
Porcupine	Nil	—	Nil	−	−	−
Llama	1	16.7%	1	+	+	+
Goat	Nil	—	2	+	+	+
Jaguar	2	28.6%	2	+	+	+
Chicken	Nil	—	Nil	−	−	−
